# Preparing public health researchers to interact with state-level legislators: evaluation of the Texas Research-to-Policy Collaboration project training

**DOI:** 10.1057/s41271-025-00555-z

**Published:** 2025-02-26

**Authors:** Tiffni Menendez, Amelia Roebuck, Shelby Flores-Thorpe, Yuzi Zhang, Kathleen Manuel, Christine Jovanovic, Alexandra van den Berg, J. Taylor Scott, D. Max Crowley, Elizabeth C. Long, Deanna M. Hoelscher

**Affiliations:** 1 Michael & Susan Dell Center for Healthy Living, Austin, TX USA; 2 School of Public Health, The University of Texas Health Science Center at Houston (UTHealth Houston), 1836 San Jacinto Blvd., Ste. 571, Austin, TX 78701 USA; 3 Department of Medicine, Institute for Minority Health Research, University of Illinois Chicago, Chicago, IL 60612 USA; 4 Edna Bennett Pierce Prevention Research Center, The Pennsylvania State University, University Park, PA USA

**Keywords:** Evidence-based policy, Health policy, Policy training, RPC, State legislature

## Abstract

To implement evidence-based health policy, public health researchers need to be equipped with the necessary skills or knowledge to engage with policymakers effectively. The study aimed to evaluate the impact of the Texas Research-to-Policy Collaboration (TX RPC) training on public health professionals and to compare the effectiveness of different training delivery modes (in-person or web-based). In the study, 59 researchers received training to increase their capacity for interacting with legislators and completed pre- and post-training surveys assessing three scales: self-efficacy, knowledge, and training needs. To determine researchers’ pre- and post-training changes, we applied paired* t* tests. Two-sample *t* tests were used to compare the differences in outcomes between the two training modes. Researchers showed significant improvement in reported policy and knowledge of the legislative process and reported reduced perceived training needs for both delivery modes. The TX RPC training prepared researchers for interacting with legislators by increasing their policy-related self-efficacy and knowledge.

## Key message

- Researcher policy capacity training should be flexible to accommodate diverse needs and constraints, including conflicting schedules, geographical location, cost, and various learning styles. Virtual training options can be a solution to these issues.
- Lectures and experiential learning activities are essential strategies for policy capacity training. Interactive, hands-on exercises in training programs can effectively enhance participants’ policy-related self-efficacy and knowledge.
- Training researchers to engage in health policy is crucial, but should be part of a comprehensive and multi-step program for effective policy change

## Introduction

The importance of basing public health policy on research, and the negative consequences of failing to do so, is manifest in our daily lives. For example, the widespread adoption of smoke-free ordinances was based on research demonstrating the harms of secondhand smoke and has protected many from negative health consequences [1, 2]. In terms of injury prevention, seatbelt legislation has saved countless lives [3]. Further, current efforts to mitigate the spread and impacts of the COVID-19 pandemic have demonstrated the importance of consistent, evidence-based health policies, and the harms that can result when research is unavailable or is not properly and consistently utilized [4, 5]. For public health-related organizations and agencies to help implement effective and evidence-based health policy, public health researchers need to understand legislator’s priorities and the legislative process, be aware of the critical time point of making policy change, and be actively engaged in policymaking [6–8]. To address these contributing factors in the translation of research to policy, the Texas Research-to-Policy Collaboration (TX RPC) Project aimed to train researchers on policy engagement so researchers could educate and collaborate with Texas legislators on important health policy issues.

There is evidence that well-designed capacity building for training scientists can improve their understanding of legislators’ competing demands and interests, skills for contacting Congressional offices, self-efficacy for dealing efficiently with unexpected issues, handling unforeseen situations, and discussing most problems without eliciting conflict [7, 9]. One study found that training of researchers is feasible, and participants reported significantly better understanding of objective and nonpartisan communication approaches, and the definition of and restrictions on lobbying [10]. Training researchers is an important step in the process of accelerating the use of research evidence but is only part of a more multi-step, comprehensive approach as detailed in both the Research-to-Policy Collaboration (RPC) and TX RPC models [7, 9, 11].

Currently, research policy trainings primarily focus on building capacity and developing partnerships between researchers and legislators. Despite this, most health policy training has been targeted to medical and nursing audiences, such as students, residents, and faculty, while a few were directed to health professionals from a broader range of health-related disciplines and backgrounds [12]. Moreover, there has been substantial variety in training venues, duration, formats, and contents, tailoring to different audiences [12, 13]. In addition to direct lectures that were often offered in training, other methods that involved interactive and experimental activities, such as case studies, mock testimonies for a hearing, and seminars, provided hands-on experience in real-world settings, were also crucial and beneficial in developing trainees’ competency to engage in policy-relevant activities [12]. The RPC training contrasts with that provided by advocacy organizations since the motivation underlying the RPC model involves supporting researchers’ nonpartisan policy engagement, training them to operate as honest brokers who partner with and support legislators’ goals without lobbying [14].

Concerning program evaluation, Heiman et al. (2016) found that the literature was limited in evaluation data for the training sessions, which impedes program comparison and identification of the best practices [12]. Among the four studies identified by Tait and Williamson that evaluated capacity before and after the training, none were conducted in the United States or at the state level with current legislative structures, which could affect training content and results [13, 15–18]. Also, no statistical analyses were conducted to compare the scores before and after training; therefore, whether there were significant differences between pre-and post-scores remains unclear. A recent study by Chrisman and Hampton (2022) assessed the confidence, competence, knowledge, importance, and intentions among public health professionals to communicate child health information with state-level legislators in Missouri before and after a single session of in-person communication training [19]. Participants were satisfied with the training and showed increases in all five outcomes, though competence was the only attribute significantly improved after training [19]. Additionally, although a pilot evaluation of RPC training previously demonstrated promising results, a gap in this literature remains due to small samples and lack of variability in the training formats tested [10].

In summary, there are limited training models that prepare researchers to engage with legislators, which perpetuates ineffective practices for bridging the communication and knowledge translation gap between researchers and legislators [20]. Additionally, there is a scarcity of planned and formal evaluations in the current literature that assess the effectiveness and efficiency of the training quantitatively and qualitatively, which hampers the identification of key components for future training. This study aims to 1) examine the effects of policy capacity training among public health researchers in Texas and 2) compare the effectiveness of two different training delivery modes on policy-related self-efficacy, knowledge, and perceived training needs to engage in state-level policymaking activities.

## Methods

### Study overview

The TX RPC Project was adapted from the federal RPC model with logistic and procedural changes to fit the Texas legislative timeline [9–11]. The RPC model has been experimentally tested and demonstrated effectiveness in bolstering the United States congressional use of research evidence in child and family bills [9]. The TX RPC Project aims to optimize the use of Texas research data and resources to assist legislators in developing effective nonpartisan health policies on a wide range of topics based on legislative priorities. Specifically, the goals of the TX RPC Project are to (1) create a bridge between public health researchers and legislative offices to build a mutually beneficial relationship around significant public health issues, (2) provide access to research, data, and resources from Texas and other relevant sources, (3) identify state policy health priorities, (4) recruit and support a network of researchers who can serve as advisors with a wide range of health-related research interests and experiences, and (5) facilitate researcher-legislator engagements and collaborations. For this project, ‘researchers’ were defined broadly to include academics, program evaluators, state agencies, and those who support the use of research in practice and communities. As a first step in the process, the TX RPC Project conducted a policy capacity training among public health researchers in Texas. The Institutional Review Board of the UTHealth Houston Texas School of Public Health Committee for the Protection of Human Subjects (HSC-SPH-19–0539) reviewed the study and determined it to be exempt for both researchers and legislators.

### Recruitment of public health researchers

Researcher recruitment began in late 2019 through established relationships with child health and public health researchers in various fields across the state. Recruitment emails to individual researchers and selected listservs were sent in September 2019, with follow-up emails in November and December 2019. Researchers were asked to fill out the intake form, online baseline survey, and register for the in-person training session held in February 2020. Researchers who received the invitation were also allowed to forward recruitment emails to other interested colleagues.

### Development of training and adaptation

Due to differences in federal and state legislative procedures, the TX RPC Project adapted the training to fit the structure of the Texas legislature with guidance from the federal RPC Project consultants [9, 11]. An Advisory Committee, consisting of members with experience with the Texas legislature, also provided input. In-person training content and activities are described in Table 1 [11, 21, 22]. Researchers could attend one of two training sessions: (1) a 1-day, 6-h in-person training in February 2020, or (2) an online training consisting of recordings of the in-person training condensed into 2 h of total viewing that could be viewed online at their own pace (Table 1), developed because of the Coronavirus Disease 2019 (COVID-19) pandemic. Resources that accompanied each research training session included detailed research expectations and information about collaborative meetings, frequently asked questions (FAQs), introspective exercises, a Texas legislative process map, tips for educating legislators and communication, legislative staff role-play exercises, and additional information on how to interact with the media on health policy. Training resources were uploaded into an online collaborative application and share drive, including a folder designated for articles and documents on communicating with legislators and a curated researcher communications protocol that consisted of email templates on how to properly address a legislator.

**Table 1:** Researcher capacity training content and web-based training duration

Session title	Activity	Duration (in min)
Project overview	Direct instruction FAQs	20
Background on effective communication with policymakers	Direct instruction FAQs	21
Interacting with policymakers	Direct instruction FAQ Introspective Exercise—participants complete a brief interpersonal assessment of their values and personal boundaries, conflict management style, and strengths and challenges, and then pair up with another participant to discuss	27
Policy process	Direct instruction FAQs	31
Advocacy vs. Lobbying	Direct instruction FAQs	2
Research communications	Direct instruction FAQs	22
Legislative meetings	Direct instruction FAQs Role-Play Exercise—mocking a meeting with a state legislator about health education in schools	6

*FAQs* Frequently asked questions

### Researcher training evaluation procedure and survey questions

To assess the researchers’ policy training needs before the in-person capacity training held in February 2020, researchers were asked to complete pre-training questions adapted from the national RPC project. A pilot study of these measures demonstrated internal consistency and suggested change sensitivity [10]. All researchers completed the pre-training assessment before the training. After the in-person training session, participants either completed the post-training evaluation at the end of training or through an online link within a week of training. For researchers who attended the online training, the post-training evaluation was completed within 2 weeks. The researcher training and evaluation timeline are shown in Table 2.

**Table 2:** TX rpc researcher policy capacity training and evaluation timeline

Date	Activity	Evaluation
September 2019	Initial researcher recruitment	Complete pre-training evaluation
November and December 2019	Researcher recruitment follow-up	Complete pre-training evaluation
February 2020	In-person training	Post-training evaluation onsite or complete within a week if the participants were not able to attend the entire in-person session
February 2020 to July 2020	Web-based training	Complete the training and post-training evaluation within a 2-week period
September 2020	Refresher webinar	N/A
October 2020	Refresher webinar	N/A

*TX RPC* Texas Research-to-Policy Collaboration

The pre-training assessment assessed the following three constructs using Likert response scales: policy-related self-efficacy, policy knowledge, and perceived training needs. The policy-related self-efficacy scale consisted of 10 questions, with responses ranging from not at all true (1 point) to exactly true (4 points). Answers were summed, with scores ranging from 10 to 40. Seven questions were included in the policy knowledge scale, with response options ranging from strongly disagree (1 point) to strongly agree (5 points), and the summed scores ranged from 7 to 35. Nine questions were asked to determine perceived training needs, with responses ranging from strongly disagree (1 point) to strongly agree (5 points), and the summed scores ranged from 9 to 45.

The post-training survey included the same scales as the pre-training survey and additional questions (Likert scale and open-ended) developed by the TX RPC investigators. Specifically, six questions were asked to evaluate the training content, format, and delivery mode, with response options ranging from very poor (1 point) to excellent (5 points); three questions were asked to determine the overall satisfaction with the training, with response options ranging from strongly disagree (1 point) to strongly agree (5 points); and one question rated the overall effectiveness of the training, and participants reported scores between 0 (less effective) and 100 (most effective). Three open-ended questions obtained qualitative feedback from researchers on the most useful and the least useful portions of the training, as well as additional comments.

### Data analysis

Frequencies and percentages were calculated for demographic information for the total sample and stratified by training delivery modes (in-person versus online). Means and standard deviations were calculated for each question and scale in the pre- and post-training survey. Paired t tests were conducted to compare policy-related self-efficacy, policy knowledge, and perceived training needs before and after researcher training. Two-sample* t* tests were used to assess differences in training measures between in-person and web-based training. Individual mean imputation was performed for missing data, where at least one item in the scale was non-missing. All data management and analyses were performed using Microsoft Excel 2007/2016 for Windows XP and Stata software version 16.0 (StataCorp LP, College Station, Texas).

## Results

Most of the participants attended the capacity training online (78%) (Table 3). Participants were predominantly female (73%), White (71%), with the highest degree of Doctor of Medicine, Juris Doctor, Doctor of Philosophy or equivalent (83%), and only 15% had never communicated with legislators or their staff during the past two years (Table 3).

**Table 3:** Demographic characteristics of training participants by training delivery mode

Characteristic	Overall (*N* = 59)	In-person (*N* = 13)	Web-based (*N* = 46)
*Gender*			
Female	43 (73%)	12 (92%)	31 (67%)
Male	16 (27%)	1 (8%)	15 (33%)
*Race/ethnicity*			
African American	1 (2%)	1 (8%)	0 (0%)
Asian	6 (10%)	0 (0%)	6 (13%)
White	42 (71%)	9 (69%)	33 (72%)
Hispanic	7 (12%)	1 (8%)	6 (13%)
multi race	3 (5%)	2 (15%)	1 (2%)
*Highest degree*			
BA/BS	2 (3%)	1 (8%)	1 (2%)
MA/MS or equivalent (MAEd, MEd, MPH, MSSW)	7 (12%)	1 (8%)	6 (13%)
MD	6 (10%)	0 (0%)	6 (13%)
JD	2 (3%)	0 (0%)	2 (4%)
PhD or equivalent (DrPH, PsyD, EdD)	40 (68%)	11 (85%)	29 (63%)
Currently in graduate school: PhD in Health Policy	1 (2%)	0 (0%)	1 (2%)
Other: MPH, JD, and PhD	1 (2%)	0 (0%)	1 (2%)
*Age*			
Under 25	2 (3%)	0 (0%)	2 (4%)
26–35	7 (12%)	2 (15%)	5 (11%)
36–45	15 (25%)	5 (38%)	10 (22%)
46–55	21 (36%)	3(23%)	18 (39%)
56–65	11 (19%)	1 (8%)	10 (22%)
> 65	3 (5%)	2 (15%)	1 (2%)
*Frequency of communication with legislative office, in the past 2 years*			
Never	9 (15%)	3 (23%)	6 (13%)
Rarely (1 or 2 times)	15 (25%)	3 (23%)	12 (26%)
Occasionally (3–5 times)	14 (24%)	4 (31%)	10 (22%)
Often (5–10 times)	12 (20%)	1 (8%)	11 (24%)
All the time (regularly, more than 10 times)	9 (15%)	2 (15%)	7 (15%)

### Comparison between pre-and post-training survey

Overall, the training resulted in increased policy self-efficacy (*p* = 0.007) and policy knowledge (*p* < 0.0001), and a decrease in training needs (*p* < 0.001) (Table 4). Both in-person and web-based training showed a significant increase in reported policy knowledge (*p* < 0.001) and a decrease in training needs (*p* = 0.006 for in-person and *p* = 0.006 for web-based). Those who attended the web-based session reported a significant increase in policy-related self-efficacy after training (*p* = 0.01). Secondary analyses further examined item-level comparisons to shed light on specific aspects of the training that might have explained the main effects seen in primary analyses (Table 5). For the overall sample, regarding policy-related self-efficacy, participants reported significant improvements in resourcefulness during meetings, developing solutions for problems during legislator meetings, and solutions for conflicts during legislator meetings. Participants reported improvements for all items included in the policy knowledge scale. For perceived training needs, participants reported fewer needs for communicating with policymakers and staff, understanding the legislative process and the role of researchers, how to work with legislative offices, responding to the needs of legislative offices, lobbying versus advocacy, and maintaining a working relationship with the offices.

**Table 4:** Difference between pre-test and post-test scale scores for the total sample and by training delivery mode, and comparisons of two training modes

	Total participants (*N* = 59)			In-person (*N* = 13)			Web-based (*N* = 46)			In-person vs. Web-based
Scales	Pre-test mean (SD)	Post-test mean (SD)	*p*-value*	Pre-test mean (SD)	Post-test mean (SD)	*p*-value*	Pre-test mean (SD)	Post-test mean (SD)	*p*-value*	*p*-value^#^
Policy-related self-efficacy ^a^	32.28 (5.48)	33.74 (4.50)	**0.007**	30.33 (4.40)	30.54 (4.70)	0.27	32.80 (5.66)	34.64 (4.05)	**0.01**	0.65
Reported policy knowledge	24.78 (5.94)	30.29 (3.64)	** < 0.001**	21.46 (6.08)	28.77 (4.34)	** < 0.001**	25.72 (5.61)	30.72 (3.35)	** < 0.001**	0.15
Training needs	33.27 (7.46)	28.37 (8.87)	**0.001**	34.31 (7.15)	30.03 (5.75)	**0.006**	32.97 (7.60)	27.90 (9.57)	**0.006**	0.66

Bold values denote statistical significance at the p<0.05 level

^*^Paired-sample test; ^#^Two-sample *t* test

^a^Sample size: policy-related self-efficacy (total, pre-test, *n* = 57; in-person, pre-test, *n* = 12; web-based, pre-test, *n* = 45), training needs (total, pre-test, *n* = 58; web-based, pre-test, *n* = 45)

**Table 5:** Difference between pre-test and post-test items for the total sample and by training delivery mode, and comparisons of two training modes

	Total participants					In-person					Web-based					In-person vs. Web-based
	Pre-test		Post-test		*p*-value*	Pre-test		Post-test		*p*-value*	Pre-test		Post-test		*p*-value*	*p*-value^#^
Items	*N*	Mean (SD)	*N*	Mean (SD)		*N*	Mean (SD)	*N*	Mean (SD)		*N*	Mean (SD)	*N*	Mean (SD)		
***Policy-related self-efficacy*** *[1* = *Not at All True, 2* = *Hardly True, 3* = *Moderately True, 4* = *Exactly True]*																
I can discuss solving difficult problems	57	3.23 (0.66)	59	3.31 (0.56)	0.25	12	2.92 (0.67)	13	2.92 (0.49)	0.67	45	3.31 (0.63)	46	3.41 (0.54)	0.29	0.98
If staff oppose me, I can find means to negotiate our different viewpoints	57	3.07 (0.70)	59	3.22 (0.56)	0.14	12	3.08 (0.51)	13	2.92 (0.49)	0.67	45	3.07 (0.75)	46	3.30 (0.55)	0.08	0.24
It is easy for me to stick to my aims and accomplish my goals	56	3.18 (0.66)	59	3.37 (0.58)	0.05	12	3.25 (0.45)	13	3.08 (0.64)	0.34	44	3.16 (0.71)	46	3.46 (0.55)	**0.03**	**0.02**
I am confident that I could deal efficiently with unexpected issues that come up	57	3.05 (0.64)	59	3.20 (0.64)	0.07	12	2.83 (0.58)	13	2.92 (0.64)	0.17	45	3.11 (0.65)	46	3.28 (0.62)	0.15	0.96
I am resourceful during meetings, which allows me to handle unforeseen situations	56	3.13 (0.76)	59	3.31 (0.65)	**0.03**	12	2.92 (0.79)	13	3 (0.58)	0.34	44	3.18 (0.76)	46	3.39 (0.65)	** < 0.05**	0.86
I can discuss most problems without eliciting conflict if I invest the necessary effort	56	3.41 (0.63)	59	3.54 (0.54)	0.09	12	3.25 (0.45)	13	3.15 (0.55)	1	44	3.45 (0.66)	46	3.65 (0.48)	0.06	0.37
I can remain calm when facing difficulties because I can rely on my coping abilities	57	3.60 (0.53)	59	3.68 (0.51)	0.20	12	3.42 (0.51)	13	3.46 (0.66)	0.44	45	3.64 (0.53)	46	3.74 (0.44)	0.33	0.70
When staff confront me with a problem, I can usually find several solutions	56	3.18 (0.74)	59	3.41 (0.65)	**0.005**	12	2.83 (0.58)	13	2.92 (0.64)	0.34	44	3.27 (0.76)	46	3.54 (0.59)	**0.009**	0.62
If I encounter conflict during discussion, I can usually think of a solution	55	3.18 (0.72)	58	3.36 (0.61)	**0.03**	12	2.83 (0.58)	13	3.08 (0.64)	**0.04**	44	3.30 (0.73)	45	3.44 (0.59)	0.15	0.45
I can usually handle whatever comes my way during conversations	55	3.27 (0.68)	59	3.34 (0.60)	0.40	12	3 (0.43)	13	3.08 (0.64)	0.17	43	3.35 (0.72)	46	3.41 (0.58)	0.66	0.57
***Reported policy knowledge*** *[1* = *Strongly Disagree, 2* = *Disagree, 3* = *Neither Agree nor Disagree, 4* = *Agree, 5* = *Strongly Agree]*																
The multiple factors that policymakers must consider when making decisions	58	4.00 (1.01)	58	4.40 (0.56)	**0.002**	13	3.69 (1.03)	13	4.31 (0.48)	**0.04**	46	4.11 (0.99)	45	4.42 (0.58)	**0.02**	0.34
The primary information sources policymakers use for decision-making	59	3.37 (1.03)	59	4.19 (0.71)	** < 0.001**	13	3 (1.0)	13	4.15 (0.80)	** < 0.001**	46	3.48 (1.03)	46	4.20 (0.69)	** < 0.001**	0.16
How policymakers’ timeframe for action differs from researchers	59	3.92 (1.04)	59	4.64 (0.55)	** < 0.001**	13	3.31 (1.03)	13	4.54 (0.52)	** < 0.001**	46	4.09 (0.98)	46	4.67 (0.56)	** < 0.001**	**0.03**
The ways in which policymakers define evidence	59	3.36 (1.06)	59	4.25 (0.66)	** < 0.001**	13	2.69 (0.95)	13	3.85 (0.90)	** < 0.001**	46	3.54 (1.03)	46	4.37 (0.53)	** < 0.001**	0.28
Common perceptions of researchers that hinder developing trusting relationships with policymakers	58	3.38 (1.11)	58	4.50 (0.54)	** < 0.001**	13	3.08 (1.12)	13	4.31 (0.48)	**0.001**	46	3.48 (1.09)	45	4.56 (0.55)	** < 0.001**	0.68
How to make contact with legislative offices	58	3.57 (1.14)	59	4.08 (0.93)	** < 0.001**	12	3.08 (1.51)	13	3.62 (1.19)	0.10	46	3.70 (1.01)	46	4.22 (0.81)	** < 0.001**	0.63
Ways I can seek to support legislative offices	57	3.18 (1.10)	59	4.20 (0.78)	** < 0.001**	11	2.55 (1.37)	13	4 (1.00)	** < 0.001**	46	3.33 (0.99)	46	4.26 (0.71)	** < 0.001**	0.06
***Training needs*** *[1* = *Strongly Disagree, 2* = *Disagree, 3* = *Neither Agree nor Disagree, 4* = *Agree, 5* = *Strongly Agree]*																
Communicating with policymakers and staff	56	3.79 (0.99)	59	3.37 (1.13)	**0.01**	13	4 (0.82)	13	3.62 (0.87)	0.05	43	3.72 (1.03)	46	3.30 (1.19)	**0.04**	0.78
Understanding the legislative process and where researchers can play a role	58	3.90 (1.12)	59	3.10 (1.24)	** < 0.001**	13	4. (1.29)	13	3.23 (0.83)	**0.03**	45	3.87 (1.08)	46	3.07 (1.34)	**0.002**	0.91
How to work with legislative offices	57	3.81 (1.06)	59	3.37 (1.16)	**0.02**	13	3.92 (0.95)	13	3.69 (1.03)	0.34	44	3.77 (1.10)	46	3.28 (1.19)	**0.04**	0.42
Best practices for synthesizing literature	58	3.12 (1.16)	59	2.85 (1.36)	0.13	13	3.08 (1.19)	13	2.77 (1.24)	0.39	45	3.13 (1.16)	46	2.87 (1.41)	0.20	0.97
Responding to needs of legislative offices	57	3.84 (0.98)	59	3.14 (1.20)	**0.001**	13	4 (0.71)	13	3.38 (1.19)	0.12	44	3.80 (1.05)	46	3.09 (1.21)	**0.005**	0.82
Regulations on lobbying and advocacy	56	3.95 (1.07)	57	3.14 (1.25)	** < 0.001**	13	3.92 (1.26)	12	3 (1.21)	**0.03**	45	3.96 (1.00)	45	3.18 (1.27)	**0.003**	0.82
Maintaining a working relationship with legislative offices	56	3.98 (1.00)	58	3.112(1.14)	** < 0.001**	13	4.23 (0.83)	13	3.46 (0.97)	**0.006**	44	3.93 (1.04)	45	3.02 (1.18)	**0.006**	0.77
Engaging with advocacy groups	56	3.34 (1.23)	59	3.08 (1.13)	0.11	13	3.77 (1.24)	13	3.31 (0.95)	0.19	43	3.21 (1.21)	46	3.02 (1.18)	0.25	0.64
Responding or engaging with media	57	3.49 (1.17)	59	3.15 (1.27)	0.11	13	3.38 (1.33)	13	3.62 (1.26)	0.57	44	3.52 (1.13)	46	3.02 (1.26)	**0.04**	0.13

Bold values denote statistical significance at the p<0.05 level

^*^Paired-sample t test; ^#^Two-sample *t* test compared the in-person with online training on the difference between pre- and post-test

### Post evaluation of satisfaction with training

Participants evaluated the training content, time allocation, quality of the materials, and in-person delivery mode as very good or excellent (Table 6). Activities during in-person training and web-based training modes were considered good to very good. Overall, participants agreed or strongly agreed that the training prepared them to participate in the TX RPC Project, met their expectations, and were satisfied with the training. On a scale of 0–100, the average training effectiveness was rated as 86.

**Table 6:** Post-training evaluation for the total sample and by training delivery mode

	Total participants		In-person		Web-based		In-person vs. Web-based
Items	*N*	Mean (SD)	*N*	Mean (SD)	*N*	Mean (SD)	*p*-value*
***Please evaluate the following aspects of today’s training:*** *[1* = *Very Poor, 2* = *Poor, 3* = *Good, 4* = *Very Good, 5* = *Excellent]*							
Content of the training	59	4.39 (0.72)	13	4.46 (0.66)	46	4.37 (0.74)	0.69
Activities during training (e.g., intro activity, introspective activity, role play)	46	3.96 (0.82)	13	4.23 (0.73)	33	3.85 (0.83)	0.15
Time allotted for the training	58	4.05 (0.80)	13	4.23 (0.73)	45	4 (0.83)	0.37
Quality of the materials presented/provided (PowerPoint, handouts)	57	4.25 (0.76)	12	4.33 (0.65)	45	4.22 (0.79)	0.66
Delivery of training—in-person on February 5. 2020	24	4.25 (0.85)	13	4.54 (0.66)	11	3.91 (0.94)	0.07
Delivery of training—via web-based recording	47	3.89 (0.79)	1	N/A	46	3.87 (0.78)	N/A
***Please rate the extent to which you agree with the following statements:*** *[1* = *Strongly Disagree, 2* = *Disagree, 3* = *Neither Agree nor Disagree, 4* = *Agree, 5* = *Strongly Agree]*							
The training prepared me to participate in the TX RPC Project	58	4.31 (0.68)	12	4.5 (0.52)	46	4.26 (0.71)	0.28
I am satisfied with the training I received today	58	4.36 (0.69)	12	4.58 (0.51)	46	4.30 (0.73)	0.22
The training met my expectations	55	4.38 (0.73)	11	4.73 (0.47)	44	4.30 (0.76)	0.08
***How would you rate today’s training overall?*** *[range: 0–100, 0* = *Least Effective, 100* = *Most Effective]*	56	85.66 (10.52)	12	90.42 (6.07)	44	84.36 (11.15)	0.08

*Two-sample *t* test

For the open-ended questions, there were 46 and 36 responses from participants on the most helpful and the least helpful portion during the training. Sixteen participants mentioned the policy process, especially the legislative procedures in Texas, and 13 mentioned that communication and interaction with legislators and their staff were the most helpful sessions. Additionally, advocacy vs. lobbying (reported by 5 participants), interactive activities (reported by 3 participants), and web-based training mode (reported by 2 participants) were cited as the most helpful sessions. The portions that were reported as less helpful centered around advocacy vs. lobbying (reported by 7 participants), web-based training delivery mode (reported by 5 participants), and interactive activities (reported by 4 participants). Some participants mentioned they would like to have more detailed information and clarification about advocacy and lobbying, while others already had training and experience on this topic. Moreover, participants commented on the technical difficulties during the web-based training, such as the clarity of audio, and the interactive activities that were unavailable via online training. Other comments expressed appreciation for the opportunity to engage and support policy activities through the TX RPC Project and the policy capacity training. Additional comments mentioned the need to include examples of researchers who had interacted with legislators in the training, using audio and visuals of speakers in the web-based training video, shortening the duration of the videos, and including quizzes.

## Discussion

This study provides an overview and initial evaluation of a policy capacity training among public health researchers. Participants reported improvements in policy-related self-efficacy and relevant knowledge and decreased perceived training needs. Also, most participants were satisfied and indicated that the training was effective. Most of the public health researchers (85%) who participated in this project had prior interactions with legislators or their staff. Since researchers were recruited on a voluntary basis, it could be that this was a self-selected sample, and that researchers that already had some experience engaging in health policy interactions were more willing to expand their skills in this area. Researchers and policymakers have different priorities and ways of interpreting and using data, so it can be difficult to bridge that divide [6]. In addition, most researchers need approval from university governmental affairs, which can limit participation. Research training models should recognize that researchers who volunteer for trainings or policy work are likely to have experience and should build on the initial learns, while seeking to recruit and engage researchers in policy topics of interest without prior experience.

Policy-related self-efficacy significantly increased from baseline to post-training. A recent review showed that communication training can increase the self-efficacy of health professionals, especially with experiential activities [23]. Interestingly, although experiential activities were included in the in-person training, the online training did not include similar exercises, yet participants showed significant increases in self-efficacy after the training. The in-person activities might need to be modified to include more effective experiential learning exercises. Training sessions that incorporate strategies such as guided practice or role model stories (whether in-person or virtual) can be one strategy to increase self-efficacy in training sessions [24].

Reported policy knowledge was improved significantly after capacity training; even when this competency broke down into specific constructs, almost all indicators remained significant. The federal RPC has found similar improvements in reported policy knowledge, specifically about lobbying regulations among researchers who received RPC capacity training [9]. Likewise, a study in Nigeria reported improvements in the policy process and capacity in the use of evidence after workshop training [16]. Features of research, such as quality, reliability, relevance, and formats, are crucial factors that determine the effective incorporation of findings within health policy. These results indicate the need for researchers to be trained in policy, as a better understanding of the political process and priorities supports and facilitates knowledge translation and utilization [25].

Overall, participants had decreased training needs after the policy capacity training. However, in the item-specific analysis, the decrease in “Best practices for synthesizing literature,” “Engaging with advocacy groups,” and “Responding or engaging with media” was not significant. The findings could be explained by the diversity in previous research training and academic backgrounds and varied prior experience of engaging with legislators among the TX RPC Project participants, as several had media experience and worked with advocacy groups in the past [11]. In addition, most public health researchers have substantial experience in synthesizing literature for academic writing in a specific field and need no further training.

The current study was conducted during the COVID-19 pandemic, which provided us with opportunities to implement the training program virtually. Our findings suggested that web-based policy capacity training could be effective and beneficial. As the researchers in the TX RPC Project are located across the state, the web-based mode overcomes the challenge of conflicting schedules and physical location. Nevertheless, considerations should be taken when implementing web-based training programs. Because the training sessions were recorded from an in-person training, participants could not engage in interactive activities, such as group discussions, role play, and FAQ sessions, and they did not receive immediate feedback if they had questions or needed clarifications. Challenges remain on how online training could best maintain participants’ engagement and commitment with minimum interpersonal interaction [26]. Fortunately, virtual training methodology has significantly improved since the COVID-19 pandemic, and these new methods would enhance the online training. When experiential learning has been incorporated into direct instruction, researchers can develop technical and interpersonal skills and competencies [10]. Therefore, conducting web-based training and including interactive activities via in-person, simulations, or synchronized virtual sessions could serve as potential strategies for both in-person and online training sessions. Another opportunity for experiential learning occurs via coaching and technical assistance provided to researchers in real time throughout the implementation of the RPC model.

Translating research into health policy can be a challenging endeavor, especially in times of uncertainty or with a lack of rigorous science. Recent learnings from the COVID-19 epidemic on a global level found varying effectiveness of implemented policies, with facial coverings and gathering restrictions providing the most effective decrease in infections and international travel controls and public information campaigns having the least effect [27]. Providing evaluation and accountability for public health policies is essential and should be incorporated into interventions to evaluate the acceleration of knowledge translation into policy, including training of researchers [28].

The current study has several strengths, including a relatively large number of public health researchers with diverse educational backgrounds, comparison of different training delivery modes, and both quantitative and qualitative evaluation. However, some limitations should be noted. First, there was no experimental control group, although there was a comparison between those participating in the in-person versus the online training sessions. Additionally, the analyses of individual questionnaire items should be interpreted cautiously since many item-level comparisons inflate the probability of Type 1 error or false positive results. Since the researcher trainings were part of a more comprehensive intervention, the individual effects of the training on health policy outcomes, e.g., bills passed, were not measured. However, the evaluation results do allow an understanding of both overall constructs as well as specific items or skills that were impacted by the training and could inform future training development as a component of a multi-step health policy effort.

## Conclusions

To support evidence-based policies through establishing trusted relationships and effective communication with legislators, researchers could benefit from trainings that prepares them with the knowledge, strategies, and confidence to be actively involved in policymaking activities. Communication between legislators and public health researchers has been difficult due to several barriers, including communication style and lack of engagement [6, 7]. The TX RPC Project policy capacity training was associated with improved researchers’ self-efficacy in communicating and interacting with legislators and staff, greater understanding of the policymaking process, and reported ability to communicate with legislators effectively. Additionally, web-based training was as effective as the in-person session and showed additional advantages for future program implementation. The current study sheds light on future policy capacity training among public health researchers and emphasizes the need to create a culture of knowledge translation that leads to increased use of research evidence in policymaking. Thus, researcher training should be an essential part of a more comprehensive, multi-step approach to accelerate knowledge translation into policy.

## Data Availability

Data available on request from the corresponding author.
